# Quality of Cattle Meat and Its Compositional Constituents

**DOI:** 10.1155/2021/7340495

**Published:** 2021-11-18

**Authors:** Umer Seid Geletu, Munera Ahmednur Usmael, Yesihak Yusuf Mummed, Abdulmuen Mohammed Ibrahim

**Affiliations:** ^1^College of Agriculture, Oda Bultum University, P.O. Box 226, Chiro, Ethiopia; ^2^Oromia Bureau Livestock and Fishery Resources, West Hararghe Zone, Chiro Wereda, P.O. Box 226, Chiro, Ethiopia; ^3^School of Animal Science and Randge Land, Haramaya University, Haramaya, Dire Dawa, Ethiopia; ^4^Regional Project Manager of L Caged Project, Haramaya University and University of Florida, Dire Dawa, Ethiopia

## Abstract

Meat is the most valuable livestock product since it is one of the main sources of protein for human consumption. Meat quality can be evaluated according to the following parameters: pH, amount of lactic acid, volatile fatty acids, bounded water, solubility of proteins, color, and tenderness. The meat composition and physical properties of muscles have been characterized for ensuring improved eating quality. Thus, the purpose of this paper was to review the major chemical compositional and physicochemical properties of meat and, at the same time, its quality attributes and factors that affect quality of meat. A number of structural features of meat as connective tissue, muscle fibers, and tendon that attaches the muscle to the bone are visible in joint meat examined through naked eyes. Water is quantitatively the most important component of meat comprising up to 75% of weight. Meat is also composed of amino acids, fatty acids, vitamins, minerals, and other important ingredients. Quality factors perceived by consumers are related to sensory attributes (e.g., color, tenderness, and flavor), nutritional properties (e.g., calories, vitamins' content, and fatty acids' profile), and appearance (e.g., exudation, marbling, and visible amount of fat). However, fresh meat quality can be defined instrumentally including composition, nutrients, color, water-holding capacity, tenderness, functionality, flavors, spoilage, and contamination. Visual inspection based on sensory quality attributes and different chemical methods are used to analyze meat quality. Other methods such as computer vision and imaging spectroscopy, gas chromatographic analysis, near-infrared technology, dual-energy X-ray absorptiometry, and computerized tomography scanning are also used in the meat industry. So, the aim of the present review is to review quality characteristics of cattle meat and its composition constituents.

## 1. Background and Justification

The general increasing human population in the world leads to the movement of people from rural areas to urban. This will increase the demand for food of livestock origin [1]. Human beings consume protein-rich foods to fulfill their nutritional requirements, mainly of animal origin, such as meat (cattle, sheep, goat, poultry, pig, fish, and seafood/shellfish), milk, and eggs [2]. Meat and meat products are most valuable livestock products and sources of high-quality protein due to their amino acid composition. They are also the major source of iron and some vitamins in the B group. So, meat consumption can alleviate several nutritional deficiencies [3].

The chemical composition and physical properties of meat determined the quality of meat (pH, color, water-holding capacity, hardness, thermal treatment losses, nutritional value of meat proteins, digestibility, etc.). These characteristics are influenced by animal species, breed, individual characteristics, sex, age, rearing technologies, and fattening, as well as other factors such as meat production procedure (feeding, transporting, and slaughtering condition) and processing (storage time or temperature condition) [4]. Composition, texture of the muscle, and some biochemical processes that take place during slaughtering, fabricating, and storing carcasses essentially affect tenderness, while the flavor, which is influenced by the fat content, can be manipulated by genetic methods, growth performance control, and dietary supplementation [5].

From the consumer perspective, quality is related to functional characteristics that range from sensory domain taste and appearance properties to cost domain storage and distribution criteria.

Among the sensory traits of meat, color, tenderness, flavor [5], and juiciness are important attributes that determine the quality of meat cuts, and consumer studies have shown that tenderness is the most important factor of the perceived eating quality of meat [6].

The consumers decide the quality of fresh meat during purchases by using its color which is an important property to determine the quality of meat. Discolored meat is related to the conversion of oxymyoglobin to metmyoglobin in the chuck and round muscle [7]. Shear force-related tenderness is an organoleptic parameter used by consumers to judge meat quality. The muscle characteristics influence tenderness of the meat during the slaughter [8] and by postmortem changes. Water-holding capacity (WHC) is an important property to determine the quality of fresh meat and meat yield. Juiciness and flavor are generally considered as being of secondary importance [9].

Quality factors perceived by consumers are tied to nutritional properties (i.e., calories, vitamins' content, and fatty acids' profile) and appearance (i.e., exudation, marbling, and amount of fat) [10]. Meat quality can be evaluated according to the maturation indexes: meat pH, amount of lactic acid, volatile fatty acids, bounded water, and solubility of proteins. Fat content and fatty acid composition of meat are becoming a quality issue that affects consumer decisions on purchase of meat [11]. The components of meat quality influenced by fatty acids are fat tissue firmness (hardness), shelf life (lipid and pigment oxidation), and flavor [12].

The meat quality determination always depends on the structure of muscle meat, including intrinsic structure (sarcomere length, myofilament diameter, and fiber types) and basic chemical composition of meat (moisture, protein, ash, and collagen content). The strongest single beef quality attribute is the marbling score, so in many countries, it is the basis for the carcass evaluation [13]. Thus, the purpose of this paper was to review meat quality based on physiochemical characteristics and chemical composition of meat.

### 1.1. General Objective

- To review on the chemical composition and physiochemical characteristics of meat quality and factors affecting the meat quality of cattle carcasses.

### 1.2. Specific Objectives

- To discuss basic meat quality attributes in terms of tenderness, flavor, pH value, water-holding capacity, marbling, and color
- To overview the basic chemical composition of cattle meat
- To review the method of analyses on the quality and composition of meat and factors that affect meat quality

## 2. Literature Review

### 2.1. Meat Composition

Meat is not only an important source of amino acids, minerals, and vitamins but also a good source of energy [14]. In terms of micronutrients, red meat is an excellent source of bioavailable iron and zinc and provides selenium and vitamins D, A, and B. It has also been proven that protein and vitamins (A and B_12_) in meat cannot be substituted by plant sources [15].

The common features of the meat structure such as connective tissue, muscle fibers, and tendon which attaches the muscle to its bone are visible in joint meat examined through the naked eye [16]. Muscle fibers are arranged in bundles surrounded by fibrous connective tissue [17], which is continuous with the tendon [18]. Endomysium is the connective tissue between individual muscle fibers. Perimysium is the sheath surrounding bundles of muscle fibers, and the connective tissue around an entire muscle is known as epimysium [17]. Individual muscles have unique connective tissue infrastructure; muscles responsible for locomotion have a greater collagen content than postural muscles, and the nature of their function contributes to the distribution of collagen within the muscle (e.g., parallel, fusiform, and pinnate) [19].

#### 2.1.1. Water

Water is quantitatively the most important component of meat comprising around 75% of weight [18] and has an important influence on color, texture, and surface appearance [10]. The water content varies inversely with the fat content, while the protein content is maintained constantly within the muscle [19]. Water-holding capacity means the ability of meat to retain tissue water present in its structure and its effects on the appearance of raw meat and its juiciness on mastication [18].

#### 2.1.2. Protein

From the nutritional and processing meat point of view, protein is the most valuable component in meat. It is the building block of the muscular tissue. Protein is a complex molecule made up of simple organic molecules known as amino acids [20]. There are 20 amino acids that are necessary for human growth and metabolism. Eight of these amino acids cannot be synthesized in our body and are described as essential, meaning that it is necessary to be contained in our diets [21]. The remaining amino acids are nonessential ones. Proteins of beef consist of essential amino acids such as leucine, isoleucine, lysine, methionine, cystine, phenylalanine, threonine, tryptophan, valine, arginine, and histidine; the last two of these are considered essential for infants [15].

#### 2.1.3. Fat

The fat portion of meat includes some fat-soluble substances, including some vitamins [14]. The fat may be present as intermuscular fat (between the muscles), intramuscular fat (marbling, i.e., within the muscles), and subcutaneous fat (under the skin/adipose tissue) [22]. Whether in the adipose tissue or muscle, fat contributes importantly to various aspects of meat quality and is central to the nutritional value of meat [23]. Fat acts as one of precursors of flavor by combining with amino acids from proteins and other components when heated [16].

Main fat properties are determined by the composition of its main component as fatty acids [5, 10]. Fatty acids are carbon chains with a methyl group at one end of the molecule and a carboxyl group at the other end. Fatty acids differ from each other in length of their hydrocarbon chains and presence or absence of double bonds. According to the degree of unsaturation, the most common dietary fatty acids have been divided into saturated fatty acids (SFA) that lack double bonds and unsaturated fatty acids (USFA) which have double bonds. USFA are subdivided into monounsaturated (MUFA) (sometimes monoenes) and polyunsaturated (polyenes) fatty acids (PUFA). MUFA have one double bond, and PUFA have two or more double bonds [24].

The fatty acid composition of adipose tissue affects its firmness because different fatty acids have different melting points. The composite fatty acids of meat melt between about 25°C and 50°C, with SFA melting at higher and PUFA at lower temperatures [24]. The total lipid content of the muscle, or intramuscular fat, has a role in the tenderness and juiciness of cooked meat. Intramuscular fat is often termed as marbling fat, although this is strictly the flecks of adipose tissue composed mainly of neutral lipid [16].

### 2.2. Meat Quality

As consumers of different goods, we always attach great importance to the quality of the products and especially to the quality of consumed food. The word “quality” can be determined differently by different people and has many meanings, and thus, it is a relative concept. However, in ISO 9000 : 2000, “quality” is defined as “the totality of features and characteristics of a product or service that bear on its ability to satisfy stated or implied needs.” [25]

Meat is one of the most important foods in the diet for the vast majority of people, particularly in the developed world [26]. Its quality, which depends on different factors, is very important for both consumers and producers. In order to produce high-quality meat, it is necessary to understand the characteristics of meat quality traits and factors to control these [27]. Aspects of meat such as source, cost, ethical factors, religion, production systems, and safety influence on the acceptability of the product by consumers.

In every case, meat quality evaluation starts with the quality control of raw meat [28]. Visual assessment of meat quality can be achieved by estimating its organoleptic attributes such as color, appearance, odor, and taste. Most sensory evaluations of meat are still performed by humans (panel test) because, currently, no laboratory analysis or mechanical device exists that can simulate all the actions of biting and chewing and that can measure or duplicate the human perceptions [19], but the fresh meat quality can be defined instrumentally including composition, nutrients, color, water-holding capacity, tenderness, functionality, flavors, spoilage, and contamination [27].

pH of meat is an important indicator of quality meat from the perspectives of its processing technologies and storage. The crucial pH value of meat (measured at approximately 24 hours after slaughter) is a direct consequence of muscle glycogen (energy) levels at slaughter. Ultimate pH of the meat determines the meat color, water-holding capacity, and texture, all of which affect the meat quality [29]. Stress of an animal before slaughtering has an important effect on pH of meat. Every animal has a certain amount of energy contained in its muscles in the form of glycogen, and once the animal is dead, muscle glycogen is converted into lactic acid that causes pH to fall [30]. The impact of nutritional factors on muscle glycogen has revealed a direct relationship between metabolizable energy intake and muscle glycogen levels [31].

Meat color is dependent on species, age, and muscle type, and the color differences are due to different contents of myoglobin in the muscle [27]. Moreover, color may be influenced by drop in pH rate and is more stable at relatively higher pH values [10] because it affects enzyme activity and the rate of oxygenation [32]. Myoglobin is the major pigment in meat [14]. The greater the concentration of myoglobin is, the darker the color of meat is [16]. Color differences of myoglobin depend on its oxygen content since myoglobin is the oxygen-carrying protein of the muscle. In the absence of oxygen, myoglobin is in the reduced form (metmyoglobin); that is why it is purple, but in the presence of oxygen, it forms oxymyoglobin which is bright red. In red meat, a bright red color associated with a high content of oxymyoglobin is a positive determinant of quality, whereas brown meat myoglobin content is a negative determinant [18].

Raw meat has little aroma, but meat flavor is developed because of several compounds produced in the postmortem muscle [10]. Abnormal odors in meat commonly happen due to the ability of meat to absorb odors from outside that develop from animal feeds, administration of drugs, fever, advanced stage of gestation, and sexual odor [33]. Moreover, meat flavor is affected by species, sex, age, stress, amount of fat, and diet of the animal [27]. True flavor develops during the cooking process and is thought to arise partly from the muscle protein reacting with some of the sugars present in the meat [6]. Flavor depends on textural characteristics, meat composition, and many other factors [5].

The amount of intramuscular fat deposited in the longissimus muscle (marbling) is a major determinant of carcass quality and predictor of palatability. Marbling is related to muscle firmness, flavor, and juiciness. It is a very significant factor in meat quality evaluation [6, 18]. It influences flavor characteristics such as juiciness and tenderness due to fatty layers, which fill meat with juice during cooking. Marbling is determined through visual observation of the carcass. Ultrasound measurements of marbling are reported as percentage intramuscular fat (%IMF) in the ribeye muscle [34].

Consumer studies have revealed that tenderness is the most important factor of meat eating quality [6, 28], and it is the most difficultly predicted one [5]. Factors that affect tenderness can be pre- and postslaughter influences [6] which include the degree of muscle contraction during rigor mortis, the amount of connective tissue, and the activity of the muscle's inherent enzyme systems [29]. Properties of beef texture include both initial (first bite with incisors) and overall tenderness (after multiple chews) and even the more complex sensory qualities of chewing and mouth feel with multiple descriptors such as fiber cohesiveness, adhesion, friability, chew count, mealiness, mushiness, softness, amount of residual connective tissue, rubberiness, and hardness [19].

Juiciness of meat is very important with respect to eating quality, and it is influenced by many factors such as ultimate pH, fat content, marination, cooking method, and degree of doneness [29].

Treatment of carcass surfaces with organic acids can have a positive result in the inhibition of microbial growth. Lactic acid (LA) is a naturally occurring acid and is used effectively by the food industry during food processing.  Table 1 is shown in detail.

Meat ranks among one of the most significant, nutritious and favored food item available to masses, which aids in fulfilling most of their body requirements. It has played a vital role in human evolution and is an imperative constituent of a well-balanced diet. It is a good source of proteins, zinc, iron, selenium, and phosphorus followed by vitamin A and B-complex vitamins. Tables 2–4 show the constituent meat in detail.

### 2.3. Factors Affecting Meat Quality

#### 2.3.1. Intrinsic Factors

The characteristics of quality meat vary among species of animal, even within more similar groups such as small ruminants [45]. On the contrary, differences in meat characteristics are assessed in sensory analyses [46]. Species-related flavors are associated with species-dependent adipose tissues, even though the acceptability of meat from different species is also linked to the population's consumption habits [47]. Breed is a clear source of variation in carcass morphology associated to fat quantity or meat quality. The impact of breed or genetic type in the lamb varies a lot. It may be stated that, as a rule, the effect of breed on instrumental and sensory meat quality, such as pH, color, texture, and sensory characteristics, is slight. Most differences are probably justified by differences in muscularity levels [48].

Gender effect (male, female, and castrated) is mainly related to the quantity of fat deposited, deposition site, growth rate, and carcass yield. Carcass attributes are more affected by gender; similarly, females are more affected than males due to their higher precociousness, whereas steers maintain an intermediate position. Differences in carcass, fat, and conformation might also affect other meat quality parameters [49]. Age and weight at slaughter also affect meat quality and are analyzed together because, taking the same genetic base, a greater weight implies a higher age, except when feed composition or level is manipulated or the animal has periods of strong feed restrictions. Fat and carcass yield proportions were affected by weight, with light lambs featuring less internal fat and lower commercial and slaughter yields but more muscle and bone percentage. Furthermore, weight affected fat contents and meat color: meat of heavy lambs was darker [50].

#### 2.3.2. Extrinsic Factors Previous to Slaughter

The feed of animals usually used has an influence on quality of color, odor, and flavor of meat, but its effects are more evident in fat [33]. The yellow color of fat due to grass feeding is common within some dairy breeds and is considered to be a negative determinant of quality [18]. Preslaughter stress of the animal also has effects on meat quality; thus, two main types of meat can be considered as aberration: dark, firm, and dry (DFD) meat and pale, soft, and exudative (PSE) meat [33]. Factors such as transportation, confinement, unfamiliar surroundings, additional handling, and deprivation of water for various periods can induce preslaughter stress [51].

Stress is an imprecise term but can be defined as an animal's response to any demand made upon it [51]. When an animal is stressed in the preslaughter environment, there is a rapid release of enzymes, cortisol and catecholamine, which may lead to depletion of glycogen, high meat ultimate pH, and dark cuts [52]. pH rises when the glycogen stores become depleted from a more prolonged stress, and lactic acid can no longer be produced [53].

Darkening of meat in the DFD condition is due to a higher respiration rate that reduces the depth of oxygen penetration and, therefore, reduces the level of visible oxymyoglobin [16]. Spoilage of DFD meat occurs more quickly than spoilage of meat with normal pH due to the absence of glucose in the tissues [54]. PSE occurred when pH of meat is less than 6 at 45 minutes after slaughter, and DFD is when ultimate pH postmortem measured after 12–48 hours is more or equal to 6. PSE meat looks pale and lean and has soft texture, low water-holding capacity, and poor functional attributes [55].

#### 2.3.3. Biochemical Changes

Muscles do not suddenly terminate all their living functions and become meat. A number of physical and chemical changes take place over a period of several hours. These include the onset of rigor mortis and the proteolytic postmortem processes [19]. In beef cattle, the conversion of muscle to meat can be a lengthy process requiring up to 48 hours or even more [19]. In particular, fresh meat quality is directly associated to muscle fiber characteristics because skeletal muscles mainly consist of muscle fibers [27].

The most significant change occurring on death is that circulation stops, and as a result, oxygen is no longer sent to the animal cells. This means that reactions begin to take place under anaerobic conditions [14]. One of the major consequences of this is that pH decreases (from ∼7.1 to ∼5.6) because in the absence of oxygen, glucose is converted into lactic acid rather than carbon dioxide and water which tenderizes the meat [14, 19].

Shortly after death, the meat appears dark and extremely firm [33]. Early postmortem, while the temperature and pH are still high, the normal level of ATP is maintained preventing actin-myosin cross-bridge formation [56, 57]. When the oxygen supply to the muscles stops, glycolysis continues anaerobically, and only two moles of ATP are produced compared to 12 moles under aerobic conditions [10, 56, 57]. Among others, ATP is produced in the muscle to drive calcium pumps and to provide energy for muscle contraction [10]. As ATP levels are reduced, Ca^2+^ levels raise and actin-myosin cross-bridges form, resulting in stiffness or rigor mortis. These rigor bonds are related with an increase in toughness [8].

The anaerobic glycolysis produces lactic acid via glycogen stores in the muscles [10, 56, 57]. Lactic acid is normally removed by the blood system, but postmortem, this removal stops, and lactic acid accumulates in the muscle causing pH to decrease, and pH will continue to decline until either the glycogen stores are used or pH is so low that the glycolysis is inhibited. The water-holding capacity is also affected by the postmortem changes mentioned above. Excessive decline of postmortem pH results in reduced WHC because pH affects the electrostatic repulsion between the filaments and thereby leads to the shrinking of myofibrils. WHC and pH are thereby correlated [57].

After slaughter, the postrigor meat is most often conditioned, also called the ageing process. During this process or period, the meat tenderness increases. The period of ageing before the meat reaches a maximum tenderness depends on species; beef needs to be conditioned for minimum ten days [57]. Tenderization during conditioning is mainly caused by changes in the myofibrillar proteins since only slight changes have been observed in the major connective tissue components such as collagen [56, 57]. pH of meat can also be related to its tenderness. The rate of pH fall along with ultimate pH has been studied though a precise relationship between tenderness and pH is not fully understood [8]. A few studies show that meat with pH below 5.3, also called PSE meat (soft, pale, and exudative), has a higher WB shear force compared to DFD meat [58]. DFD meat stands for dark firm dry and has pH above 6.0. Other studies have found that peak/shear force was lowest at pH 5.4, then increased until pH 5.8–6.0, and then decreased again [59, 60].

When the ageing time increased, the difference between WB peak forces between different ultimate pH became smaller [59]. A day or so later, meat is lighter in color and is moist. Over 30% of fluid can be squeezed from it. Protein synthesis and degradation rates in the live animal may influence tenderization postmortem, levels of stored glycogen may influence the rate and extent of postmortem glycolysis, and levels of fat in all the depots may influence the rate of temperature decline postmortem, thus affecting rigor development and proteolytic enzyme activities [19].

#### 2.3.4. Method of Evaluation of Meat Quality and Composition

Meat quality is most often defined as fresh meat eating quality which describes meat for fresh meat consumption and technological quality which describes meat for further processing [64]. The main quality parameters of technological quality are water-holding capacity, color, and texture of fat [57]. Eating quality parameters are mainly characterized by texture, juiciness, and flavor/odour [57, 64] although for many consumers, fat content is an important factor. For the slaughterhouse, the most important meat quality parameters are lean meat and the fat percentage. Visual inspection has been serving the meat industry for many years, but it may lead to inconsistencies and variations despite the professional training of the graders [65].

Traditionally, sensory quality attributes are inspected by well-trained assessors. In some abattoirs, tenderness is evaluated using a “finger method.” Meat color and marbling evaluation methods are similar and are usually carried out by comparing ribeye muscle color or the proportion of intramuscular fat within it against reference standards specific for each of the meat species [66]. Computer vision and imaging spectroscopy are proven technologies that have the capacity to deliver consistent, speedy, and affordable quality assessments. Computer vision mostly uses the reflectance mode to detect external quality characteristics such as color, size, shape, and texture [65].

Cattle carcasses are classified either manually or automatically using the EUROP grading system. Manual classification is carried out visually by a trained operator at the slaughterhouse, whereas different systems are developed for automatic classification; these are all based on video image analysis (VIA) [67]. There are numerous methods of measuring meat composition; most of them are both time-consuming and expensive. Measuring fresh meat quality by chemical methods is possible though it is time-consuming and therefore not an option due to shelf life and high cost. Fat quality is therefore most often measured by the level of saturation, either by determining the amount of iodine (iodine number) [57] or by gas chromatographic analysis (determines the fatty acid composition) [68]. Also, physical properties such as melting point and color can be measured [68].

There are other methods to measure meat quality than by chemical analysis. Instruments available only measure a few parameters, but most often, more quality parameter is of interest. CFS MultiTrack is integrated into the grinder and measures fat and lean meat percentage by near-infrared (NIR) technology during mincing [69]. The method of NIR has shown to be able to predict meat tenderness (Warner-Bratzler shear force), protein, moisture, and fat content [70].

Another piece of equipment commercially available is the MeatMaster from Foss which not only scans (X-ray) and estimates the fat content of raw meat but also spots metal and bone [71]. In different studies, dual-energy X-ray absorptiometry (DEXA) has demonstrated that the method is able to predict carcass and tenderness in beef striploins [72]. Another piece of X-ray equipment is computerized tomography (CT) that has proved to be more precise and reliable predicting carcass composition than manual dissection in lamb carcasses [73].

The shear force-deformation curves were obtained; for cooked meat, using a Warner-Bratzler (WB) shear device has suggested that (a) initial yield force values reflected, primarily, the strength of the myofibrillar structure and (b) the difference between initial yield and peak force values was greatly influenced by the mechanical properties of the connective tissue structure. This interpretation was, obviously, simplistic but appeared to accord well with the results obtained using samples subjected to a variety of different treatments including cooking, myofibrillar contraction state, ageing, and, to a limited extent, animal age. There is thus some evidence that the connective tissue contribution to initial yield force values is probably small. Once cooked steak has cooled off, the scientists collect six to eight core samples, each half an inch (1.27 cm) in diameter. They measure the pounds or kilograms of force required to shear the cores completely in half using a steel blade specifically designed to mimic the action of the human jaw. The mean for all the cores is the shear force for the animal. On the Warner-Bratzler system, beef tenderloin typically has a shear force of around 5.7 lbs (2.6 kg). A top round steak has a shear force of around 11.7 lbs (5.3 kg).

## 3. Conclusions and Recommendations

Since meat is an important source of essential nutrients, its quality is very important for both consumers and producers, and it depends on different factors. The review shows that quality factors perceived by consumers are related to sensory characteristics such as color, tenderness, and flavor, nutritional properties such as calories, vitamins' content, and fatty acids' profile, and appearance such as exudation, marbling, and visible amount of fat. Fresh meat quality can also be defined instrumentally by scientific factors including composition, nutrients, pH, color, water-holding capacity, tenderness, functionality, flavors, spoilage, and contamination. There are numerous methods of measuring meat quality and composition. All the agents within the meat chain from the farm to fork should be evaluated so that quality meat and meat products in terms of durability and acceptability could be obtained (Tables 1–4).

## Figures and Tables

**Table 1:** Effects on meat quality traits by lactic and/or acetic acid application [35–37].

Acid	Appl. conc.		Substrate	Changes	Reference
A	S	2%	Beef carcass	Col.	Osthold et al. [38]
L	S	1%	Beef carcass	Col.	Osthold et al. [38]
L	S	1.25%	Veal carcass	None	Smulders and Woolthuis (1985)
L	S	1%	Pork carcass	None	Prasai et al. [39]
L	S	2.4%	Pork carcass∗	Col.	Labots et al. [40]
A	S	2%	Pork loin	Col.	Cacciarelli et al. [41]
A/L/M	S	1%	Beefsteak	None	Dixon et al. [42]
A/L/M	S	1%	Beef striploins	None	Acuff et al. [43]

L: lactic acid; A: acetic acid; M: mix of lactic and acetic acid; (): reversible changes; ^*∗*^ applies only to meat surfaces and surfaces of body cavities; I: immersion; S: spray; Col.: color. Source: [44].

**Table 2:** Nutritional composition of meat.

Essential amino acids				
Amino acids	Category	Beef	Lamb	Pork
Lysine	Essential	8.2	7.5	7.9
Leucine	Essential	8.5	7.2	7.6
Isoleucine	Essential	5.0	4.7	4.8
Cystine	Essential	1.5	1.5	1.2
Threonine	Essential	4.2	4.8	5.2
Methionine	Essential	2.2	2.4	2.6
Tryptophan	Essential	1.3	1.2	1.5
Phenylalanine	Essential	4.1	3.8	4.3
Arginine	Essential	6.4	6.8	6.6
Histidine	Essential	2.8	2.9	3.1
Valine	Essential	5.6	5.1	5.2

Nonessential amino acids				
Amino acid	Category	Beef	Lamb	Pork
Proline	Nonessential	5.2	4.7	4.4
Glutamic acid	Nonessential	14.3	14.5	14.6
Aspartic acid	Nonessential	8.9	8.6	8.8
Glycine	Nonessential	7.2	6.8	6.0
Tyrosine	Nonessential	3.3	3.3	3.1
Serine	Nonessential	3.9	3.8	4.1
Alanine	Nonessential	6.3	6.2	6.4

Source: [61].

**Table 3:** Mineral contents (mg/100 g) of meat and meat products.

Meat source	K	Cu	Fe	P	Zn	Mg	Na	Ca
Chopped mutton (raw)	244	0.15	0.99	174	4.2	18.8	74	12.5
Chopped mutton (grilled)	303	0.25	2.5	205	4.2	22.7	101	17.9
Beefsteak (raw)	335	0.1	2.4	275	4.2	24.4	68	5.5
Beefsteak (grilled)	369	0.22	3.8	302	5.8	25.1	66	901

Source: [62].

**Table 4:** Vitamin content of various raw meat.

Vitamin units/100 g raw meat	Beef	Bacon	Mutton	Veal	Pork
A (inter. unit.)	Trace	Trace	Trace	Trace	Trace
D (inter. unit.)	Trace	Trace	Trace	Trace	Trace
B_1_ (mg)	0.06	0.39	0.14	0.11	1.2
B_2_ (mg)	0.21	0.16	0.24	0.26	0.21
Nicotinic acid (mg)	5.1	1.6	4.99	7.1	5.2
Pantothenic acid (mg)	0.5	0.4	0.6	0.5	0.5
Biotin (*μ*g)	2	8	4	6	5
Folic acid (*μ*g)	9	Nil	2	6	2
B_6_ (mg)	0.2	0.3	0.3	0.4	0.4
B_12_ (*μ*g)	2	Nil	2	Nil	2
C (mg)	Nil	Nil	Nil	Nil	Nil

Source: [63].

## Data Availability

The data used to support the findings of this study are available from the corresponding author upon request.
